# Common and rare variants in patients with early onset drusen maculopathy

**DOI:** 10.1111/cge.14212

**Published:** 2022-09-13

**Authors:** Anita de Breuk, Yara T. E. Lechanteur, Galuh Astuti, Jordi Corominas Galbany, Caroline C. W. Klaver, Carel B. Hoyng, Anneke I. den Hollander

**Affiliations:** ^1^ Department of Ophthalmology, Donders Institute for Brain, Cognition and Behaviour Radboud University Medical Center Nijmegen The Netherlands; ^2^ Department of Human Genetics Radboud University Medical Center Nijmegen The Netherlands; ^3^ Division of Human Genetics, Center for Biomedical Research, Faculty of Medicine Diponegoro University Semarang Indonesia; ^4^ Department of Ophthalmology, Department of Epidemiology Erasmus Medical Center Rotterdam The Netherlands; ^5^ Institute of Molecular and Clinical Ophthalmology Basel Switzerland; ^6^ Present address: AbbVie, Genomics Research Center Cambridge Massachusetts USA

**Keywords:** age‐related macular degeneration, complement pathway, early onset drusen maculopathy, genetic variation, lipid metabolism, whole genome sequencing

## Abstract

Early onset drusen maculopathy (EODM) can lead to advanced macular degeneration at a young age, affecting quality of life. However, the genetic causes of EODM are not well studied. We performed whole genome sequencing in 49 EODM patients. Common genetic variants were analysed by calculating genetic risk scores based on 52 age‐related macular generation (AMD)‐associated variants, and we analysed rare variants in candidate genes to identify potential deleterious variants that might contribute to EODM development. We demonstrate that the 52 AMD‐associated variants contributed to EODM, especially variants located in the complement pathway. Furthermore, we identified 26 rare genetic variants predicted to be pathogenic based on in silico prediction tools or based on reported pathogenicity in literature. These variants are located predominantly in the complement and lipid metabolism pathways. Last, evaluation of 18 genes causing inherited retinal dystrophies that can mimic AMD characteristics, revealed 11 potential deleterious variants in eight EODM patients. However, phenotypic characteristics did not point towards a retinal dystrophy in these patients. In conclusion, this study reports new insights into rare variants that are potentially involved in EODM development, and which are relevant for future studies unravelling the aetiology of EODM.

## Graphical abstract

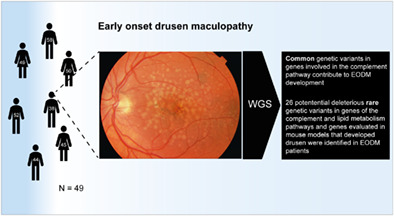

## 1 INTRODUCTION

Age‐related macular degeneration (AMD) is one of the leading causes of severe visual impairment among elderly globally.^1^ Genetic predisposition in combination with ageing and several environmental factors underlie the disease aetiology. In a recent genome‐wide association study (GWAS), including ~16 000 AMD cases and ~17 000 control individuals, 52 genetic variants are found to be independently associated with AMD, including 45 common and seven rare variants.^2^ The complement pathway is considered the most important pathway involved in AMD, as 19/52 risk increasing and protective variants are located in or near genes of the complement pathway.^2^ In addition, a collective enrichment of rare variants in complement genes has been identified in AMD case–control and family studies,^3^, ^4^ however, due to their low allele frequency, only for a limited number of individual rare variants a robust association could be established.^2^, ^5^, ^6^

Although AMD mainly affects the elderly, some individuals develop AMD characteristics, including drusen, which are deposits that accumulate underneath the retinal pigment epithelium (RPE), RPE alterations, geographic atrophy (GA) and choroidal neovascularization (CNV) at a much younger age. Consequently, these patients with early onset drusen maculopathy (EODM) often develop the vision‐threatening end stages of the disease, including GA and CNV, earlier in life. Its early onset suggests a prominent role for genetic factors rather than environmental factors in EODM development. In particular rare, highly penetrant variants may contribute to an early onset of AMD characteristics.^7^, ^8^, ^9^, ^10^ Recently, we evaluated a large cohort of 89 EODM patients, and identified rare variants in complement factor H (*CFH*) in ~30% of EODM patients.^11^ We hypothesised that in EODM patients without a potential causative rare variant in *CFH*, rare variants in other genes might contribute to EODM. In addition, common AMD‐associated variants may also contribute to EODM, as we hypothesise that EODM is an early manifestation of AMD, caused by a combination of common variants and highly penetrant rare variants. Caution is warranted when studying EODM, as phenotypically some inherited retinal dystrophies (IRDs) resemble EODM, for example, Stargardt disease (characterised by yellow flecks, which are different from drusen, and GA) and Sorsby fundus dystrophy (SFD, characterised by yellow, subretinal, drusen‐like deposits and CNV).

The aim of this study is to determine the contribution of common AMD‐associated variants, and to identify potential disease‐causing rare variants in patients with EODM.

## 2 MATERIALS AND METHODS

### 2.1 Study population

We collected a cohort of 89 unrelated patients with EODM from the European Genetic Database (EUGENDA), as described previously,^11^ and one EODM patient from the Institut de la Màcula, Barcelona. EODM was defined as any sign of age‐related maculopathy diagnosed ≤55 years of age, or severe signs of age‐related maculopathy diagnosed between 56 and 65 years of age. In this current study, we selected 49 patients from the EODM cohort in whom no rare, potential causative variants in the complement genes *CFH, CFI, C3, C9* and *CFB* were identified.^11^ All except one of the EODM patients were from European ancestry. Colour fundus photographs of EODM patients were graded by experienced graders from EyeNED Reading Center under supervision of a senior specialist according to the international grading system based on the Wisconsin Age‐Related Maculopathy Grading System, and reclassified into the Rotterdam Classification, as described previously.^12^, ^13^, ^14^ In addition, a case–control cohort from the EUGENDA database, including 925 control individuals without AMD ≥65 years of age, 577 early/intermediate AMD patients, and 1155 advanced AMD patients, was used as reference for the genetic risk score (GRS) distribution. Grading of the reference cohort was performed according to the CIRCL grading protocol, as described previously.^15^ This research was approved by ethical committees at the Radboud university medical center, Nijmegen, The Netherlands, the University Hospital of Cologne, Cologne, Germany, and the Institut de la Màcula, Barcelona, Spain, and adhered to the tenets of the Declaration of Helsinki, and all study participants provided written informed consent.

### 2.2 Whole genome sequencing

WGS was performed on a BGISeq500 using 2 × 150 bp paired‐end reads and a 30‐fold minimal median coverage per genome by BGI Genomics. The sequencing reads were mapped to the human reference genome (hg19) using the Burrows‐Wheeler Aligner (BWA) v0.7.13. Qualimap V.2.2.1 was applied to assess quality of sequencing alignment data based on insert size, percentage mapped reads, percentage duplicated mapped reads, coverage, bases with >20× coverage and error rate. Variant calling was performed using xAtlas V.0.1 and thereafter annotated using the Variant Effect Predictor (VEP V.91) and Gencode V.34lift37 basic gene annotations. Single nucleotide variants were annotated using an in‐house developed pipeline which provides further information including different population allele frequency databases (e.g. gnomAD [https://gnomad.broadinstitute.org], GoNL, Wellderly, 1000genomes, in‐house variant frequencies database containing WES‐data of 15 576 individuals), in silico prediction (e.g. Grantham, PhyloP, CADD_PHRED, SpliceAI), predicted protein effect, gene and disease OMIM description, gene regulation and expression data.

### 2.3 Genetic risk scores

The 52 AMD‐associated variants, as reported previously,^2^ were extracted from the WGS dataset (Table S1). Subsequently, an overall GRS (including all 52 variants), a complement GRS (including 19/52 complement‐related variants), and a lipid GRS (including 7/52 lipid‐related variants) were calculated based on the formula: $\mathrm{GRS}=\sum_{i=1}^{52}\left(G_{i}\beta_{i}\right)$. $G_{i}$ represents the genotype of variant i, coded as 0, 1 of 2, based on the number of minor alleles, and $\beta_{i}$ represents the effect size of variant i (natural logarithm of the fully conditioned odds ratio of the minor allele of variant i) based on the GWAS of the International Age‐related Macular Degeneration Genomics Consortium.^2^

### 2.4 Filtering of the rare variants

We performed multiple filtering steps to identify potential disease‐causing variants in EODM patients. As a first step, WGS data were filtered on SNVs with a minor allele frequency (MAF) <1%, based on the in‐house database, and on the Genome Aggregation Database (gnomAD). Next, we extracted rare variants present in four candidate gene lists, including genes involved in the complement pathway, the lipid pathway, genes evaluated in mouse models that developed drusen or other AMD characteristics, and genes located in the 34 AMD‐associated loci.^2^, ^16^, ^17^, ^18^, ^19^, ^20^ In addition, we filtered for rare variants in 18 genes associated with autosomal dominant (AD) and autosomal recessive (AR) IRDs that can mimic AMD.^21^, ^22^ Details of the gene lists are provided in Table S2. Following extraction of rare variants in candidate genes, we prioritised the rare variants based on variant type and in silico prediction tools (Combined Annotation Dependent Depletion [CADD], threshold 15; PhyloP, threshold 2.7; Grantham score, threshold 80). As a last step, we evaluated reported pathogenicity based on ClinVar.^23^ For the 18 IRD genes, we screened for a set of (likely) pathogenic intronic variants, structural variants and copy number variants previously described in these 18 genes by using the Leiden Open Variation Database (LOVD) version 3.0, and previous literature.^24^, ^25^

### 2.5 Statistical analysis

Continuous variables that were normally distributed were displayed as means with corresponding standard deviations (SD), and medians with corresponding interquartile ranges (IQR) were used for skewed distributions. Categorical variables were displayed as proportions with corresponding percentages. GRS, complement GRS and lipid GRS among disease stages were compared using Kruskal–Wallis tests for skewed data and one‐way analysis of variance for normally distributed data. Statistical analysis and data visualisation were performed with GraphPad Prism version 5.03 for Windows, GraphPad Software, San Diego, California USA, www.graphpad.com.

## 3 RESULTS

### 3.1 Description of the EODM cohort

The 49 EOMD patients in this current study had a mean (SD) age of 51.8 (9.2) years, and 67.3% were female. 55.1% showed signs of early/intermediate AMD (RC 1–3), and 44.9% were affected by advanced disease stages, including GA (*n* = 13), CNV (*n* = 8), or a combination of GA and CNV (*n* = 1) (Table 1 **)**.

**TABLE 1:** General characteristics of the EODM study cohort

Characteristic	EODM (*N* = 49)
Age, mean (SD), years	51.8 (9.2)
Age at diagnosis, median (IQR), years	48.0 (44.0–56.0)
Gender, *n* (%)	
Male	16 (32.7)
Female	33 (67.3)
Disease stage, *n* (%)	
Stage 1 or 2 ARM	10 (20.4)
Stage 3 ARM	17 (34.7)
Stage 4 ARM	
Geographic atrophy	13 (26.5)
Choroidal neovascularization	8 (16.3)
Mixed (geographic atrophy + choroidal neovascularization)	1 (2.0)
Family history of AMD, *n* (%)	
Yes	12 (24.5)
No	15 (30.6)
Unknown	22 (44.9)
Smoking, *n* (%)	
Never smoked	18 (36.7)
Former smoker	15 (30.6)
Current smoker	9 (18.4)
Unknown	7 (14.3)

*Note*: General characteristics of the 49 patients with early onset drusen maculopathy, included in this study. Disease stage is based on the Rotterdam Classification, as described previously.^14^

Abbreviations: AMD, age‐related macular degeneration; ARM, age‐related maculopathy; EODM, early onset drusen maculopathy.

### 3.2 Contribution of common AMD‐associated variants to EODM

**FIGURE 1:**
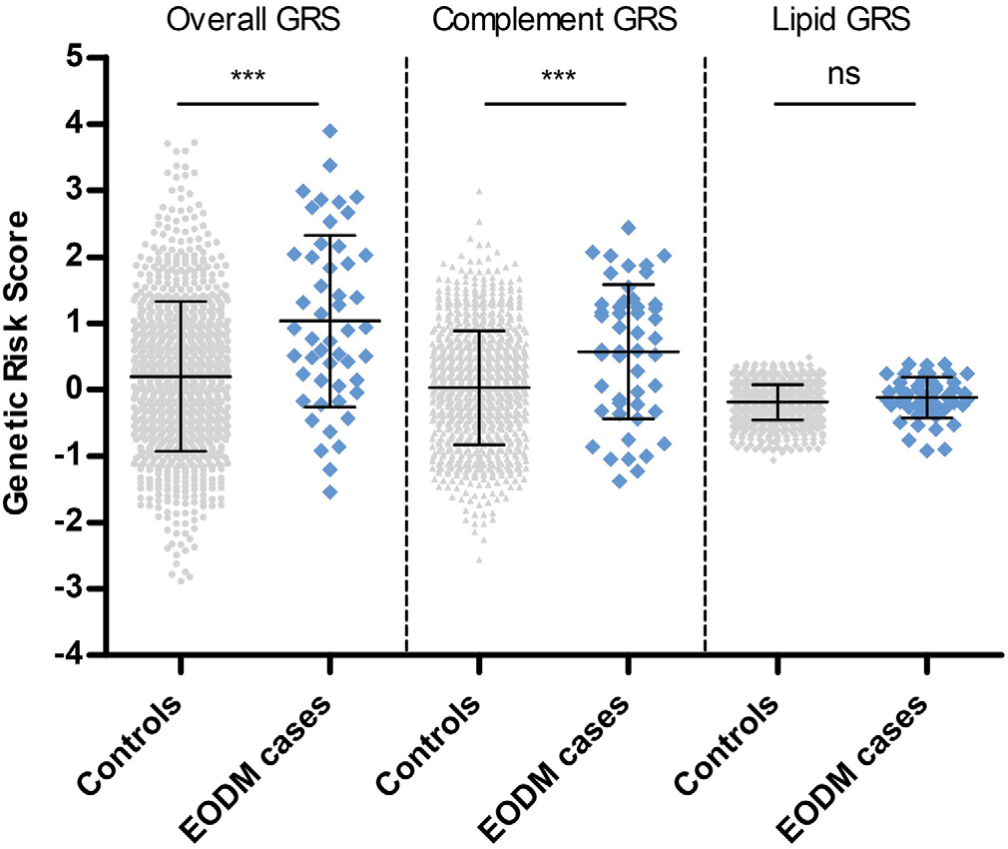
Contribution of AMD‐associated variants to EODM. Mean GRS, complement GRS, and lipid GRS in EODM cases (blue) and control individuals (grey). Error bars: mean ± SD. ***p < 0.001. GRS, genetic risk score; ns, not significant [Colour figure can be viewed at wileyonlinelibrary.com]

First, we determined whether AMD‐associated variants contribute to EODM, by comparing GRSs of 49 EODM patients to GRSs of 925 control individuals from the AMD case–control reference cohort. The mean (SD) GRS was higher in EODM compared to control individuals (1.03 (1.29) vs 0.20 (1.13), *p* < 0.001), indicating that the AMD‐associated variants contribute to EODM development. Considering that the overall GRS consists of genetic variants present in different disease pathways, we extracted the complement and lipid variants to assess pathway specific GRSs. We observed a higher mean (SD) complement GRS in EODM cases compared to control individuals (0.57 (1.01) vs. 0.03 (0.86), *p* < 0.001). In contrast to the complement GRS, the lipid GRS did not differ between EODM cases and control individuals (−0.12 (0.31) vs. −0.19 (0.27), *p* = 0.08) (Figure 1).

**FIGURE 2:**
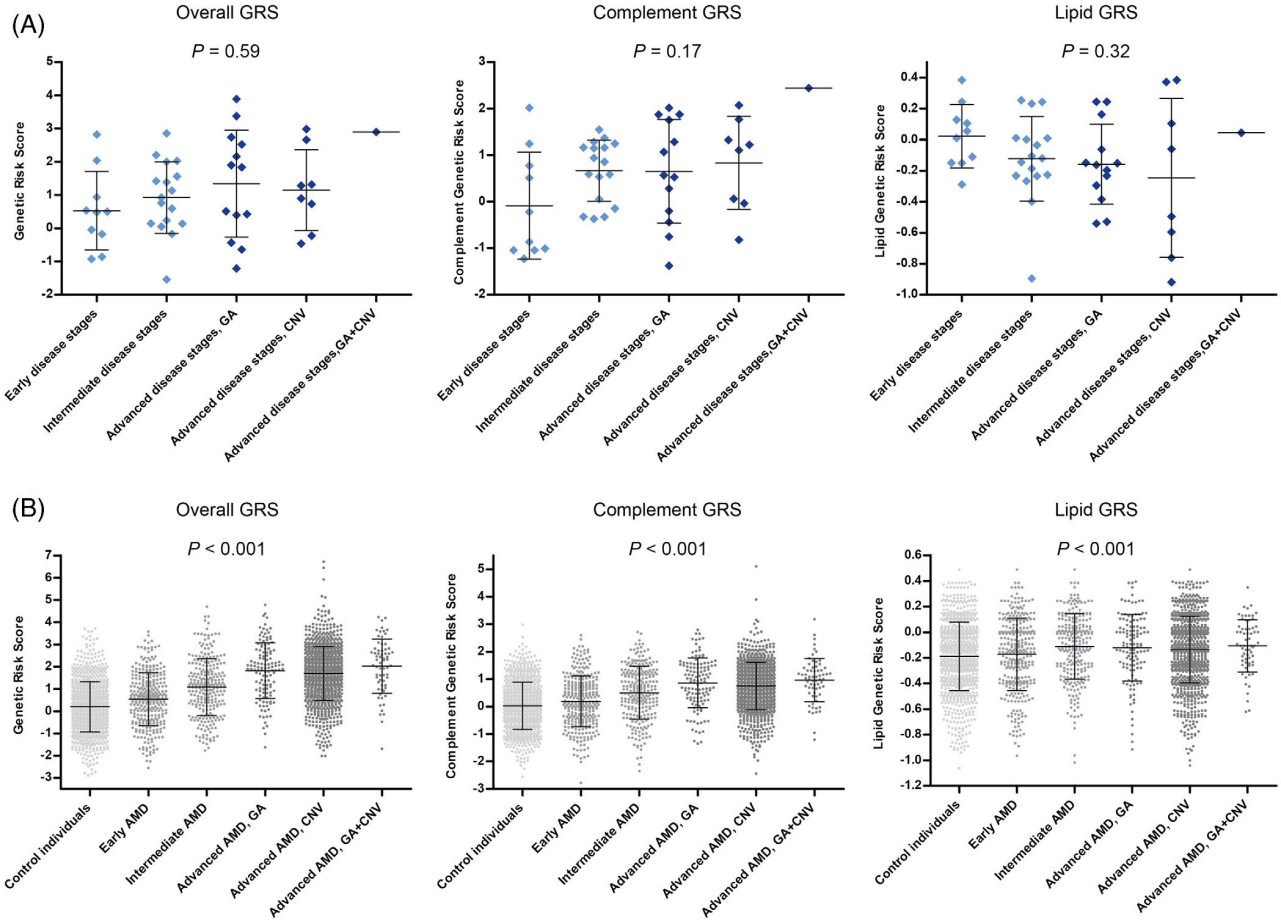
GRS distribution among disease stages. GRS, complement GRS, and lipid GRS among disease stages in EODM patients (A) and in the AMD case–control reference cohort (B). Error bars: mean ± SD. AMD, age‐related macular degeneration; CNV, choroidal neovascularization; GA, geographic atrophy; GRS, genetic risk score [Colour figure can be viewed at wileyonlinelibrary.com]

In addition, we evaluated GRSs among the different disease stages. In the AMD case–control reference cohort a significant increase in mean GRS, complement GRS and lipid GRS was observed with increasing severity of the disease stages (*p* < 0.001 for all). In EODM, overall GRS and complement GRS showed a trend of increasing GRS and complement GRS, with increasing severity of disease stages (*p* = 0.59 and *p* = 0.17, respectively). The lipid GRS showed a trend of decreasing lipid GRS, with increasing severity of disease stages (*p* = 0.32) (Figure 2). GRSs per patient are summarised in Table S3.

### 3.3 Potential deleterious rare variants in candidate genes in EODM

Next, we studied the contribution of rare variants in EODM. We first focused on candidate genes present in the complement gene list, lipid gene list and list of genes tested in mouse models that developed drusen or other AMD characteristics. In total, 97 unique candidate genes were present in those three candidate gene lists (Table S2), and we identified 51 genes that harboured a rare variant (not necessarily the same variant) in at least two EODM patients (Table S4 sections A–C). Of note, 5/51 genes (*C3, CFH, ABOB, APOE, LDLR*) were present in two candidate gene lists.

Out of all variants in those 51 genes, 26 rare variants in 19 genes were considered to be potentially deleterious based on variant type (nonsense, frameshift, stop‐loss, canonical splice‐site), in silico prediction tools (PhyloP, CADD and Grantham score above the threshold) or pathogenicity category according to ClinVar (pathogenic or likely pathogenic) (Table 2). Most of these rare variants (16/26) were located in complement‐related genes (*C3, CFH, CFHR2, C8A, C8B, C9, CSMD1, CSMD2, FCN1, MASP1, MASP2*). Two of these variants were located in *C3*: c.1661A > G (p.Asp554Gly) is predicted to be damaging based on all three in silico prediction tools, and c.4992A > G (p.Ter1664TrpextTer24) results in a stop‐loss. One frameshift variant (c.454del [p.Ala152Glnfs*8]) and two nonsense variants (c.865A > T (p.Lys289*) and c.162C > A (p.Cys54*)) in *C8A, C8B* and *C9*, respectively, were identified in three different EODM patients. Furthermore, a rare splice‐site variant in *CFH* (c.350 + 6 T > G), reported to be pathogenic in ClinVar, and a rare nonsense variant in *CFHR2* (c.595G > T [p.Glu199*]) were identified in two different EODM patients. The other potential deleterious rare variants in complement‐related genes *CSMD1, CSMD2, FCN1, MASP1* and *MASP2* had either a high CADD score, or were potentially deleterious based on variant type, including a nonsense variant in *FCN1* (c.868C > T [p.Arg290*]) and a frameshift variant in *MASP1* (c.1770del [p.Lys591Serfs*11]). However, these variants were not reported in ClinVar, except for the variant in *MASP2*, which was reported as (likely) benign. The other potential deleterious rare variants were identified in lipid‐related genes (7/26; *LDLR, APOB, MTTP, CYP4V2, ABCA1, ACAT1*) or in genes that were evaluated in mouse models (8/26; *LDLR, APOB*, *C3, CFH, CX3CR1, CD36*).

**TABLE 2:** Potential damaging rare variants in candidate genes in EODM patients

Candidate gene list	Gene	Variant	Type of variant	PhyloP	CADD	Grantham score	Pathogenicity (as reported in ClinVar)	No. of EODM patients (MAF %)	gnomAD v2.1.1. (MAF, %)
Lipid & mouse models	*LDLR*	c.47 T > C (p.Leu16Pro)	Missense	3.1	24.2	98	VUS	2 (2.04%)	NA
	*APOB*	c.3449 T > A (p.Met1150Lys)	Missense	3.3	23.4	95	VUS	1 (1.02%)	0.002
		c.2437‐2A > G	Splice‐site	2.6	25.4	0	VUS	1 (1.02%)	NA
Complement and mouse models	*C3*	c.4992A > G (p.*1664Trpext*58)	Stop‐loss	4.6	14.4	1000	NA	1 (1.02%)	NA
		c.1661A > G (p.Asp554Gly)	Missense	7.3	25.1	94	NA	1 (1.02%)	NA
	*CFH*	c.350 + 6 T > G	Splice‐site	2.7	20.4	0	Pathogenic	1 (1.02%)	NA
Mouse models	*CX3CR1*	c.74A > G (p.Asp25Gly)	Missense	5.4	22.0	94	Likely benign	1 (1.02%)	0.16
	*CD36*	c.1144C > T (p.Gln382*)	Nonsense	0.6	47.0	1000	VUS^a^	1 (1.02%)	0.01
Lipid	*MTTP*	c.103_106del (p.Lys35Valfs*20)	Frameshift	−0.2	9.9	1000	NA	1 (1.02%)	NA
	*CYP4V2*	c.169 T > C (p.Tyr57His)	Missense	4.6	23.9	83	VUS	1 (1.02%)	0.02
	*ABCA1*	c.3542C > T (p.Ser1181Phe)	Missense	9.5	23.9	155	VUS	1 (1.02%)	0.14
	*ACAT1*	c.1217A > G (p.Glu406Gly)	Missense	9.0	32.0	98	VUS	2 (2.04%)	0.03
Complement	*CFHR2*	c.595G > T (p.Glu199*)	Nonsense	−1.4	35.0	1000	Benign	1 (1.02%)	0.75
	*C8A*	c.454del (p.Ala152Glnfs*8)	Frameshift	−100.0	14.7	0	NA	1 (1.02%)	0.0004
	*C8B*	c.865A > T (p.Lys289*)	Nonsense	1.2	39.0	1000	NA	1 (1.02%)	NA
	*C9*	c.162C > A (p.Cys54*)	Nonsense	1.3	35.0	1000	Pathogenic/likely pathogenic	1 (1.02%)	0.09
	*CSMD1*	c.8687C > T (p.Thr2896Met)	Missense	5.3	22.5	81	NA	1 (1.02%)	0.004
		c.554G > T (p.Cys185Phe)	Missense	7.6	26.2	205	NA	1 (1.02%)	NA
	*CSMD2*	c.6073C > T (p.Arg2025Trp)	Missense	5.2	32.0	101	NA	1 (1.02%)	0.002
		c.3839C > T (p.Ser1280Leu)	Missense	3.5	21.5	145	NA	1 (1.02%)	0.005
		c.3520G > A (p.Gly1174Arg)	Missense	4.0	24.5	125	NA	1 (1.02%)	0.10
		c.1394G > A (p.Gly465Asp)	Missense	7.5	26.8	94	NA	1 (1.02%)	0.05
	*FCN1*	c.868C > T (p.Arg290*)	Nonsense	−2.5	0.0	1000	NA	1 (1.02%)	NA
	*MASP1*	c.1770del (p.Lys591Serfs*11)	Frameshift	−100.0	10.1	0	NA	1 (1.02%)	0.02
	*MASP2*	c.467G > A (p.Cys156Tyr)	Missense	7.6	26.5	194	Benign/likely benign	1 (1.02%)	0.59

*Note*: Rare potentially deleterious variants in EODM patients in genes involved in the complement pathway, lipid pathway, or in genes tested in mouse models that developed drusen or other AMD characteristics.

Abbreviations: CADD, combined annotation dependent depletion; EODM, early onset drusen maculopathy; MAF, minor allele frequency; VUS, variant of uncertain significance.

^a^ Originally reported as likely pathogenic in ClinVar, however, without any evidence of phenotype of the individual carrying this variant.

### 3.4 Overlapping rare variants identified in at least two EODM patients

**FIGURE 3:**
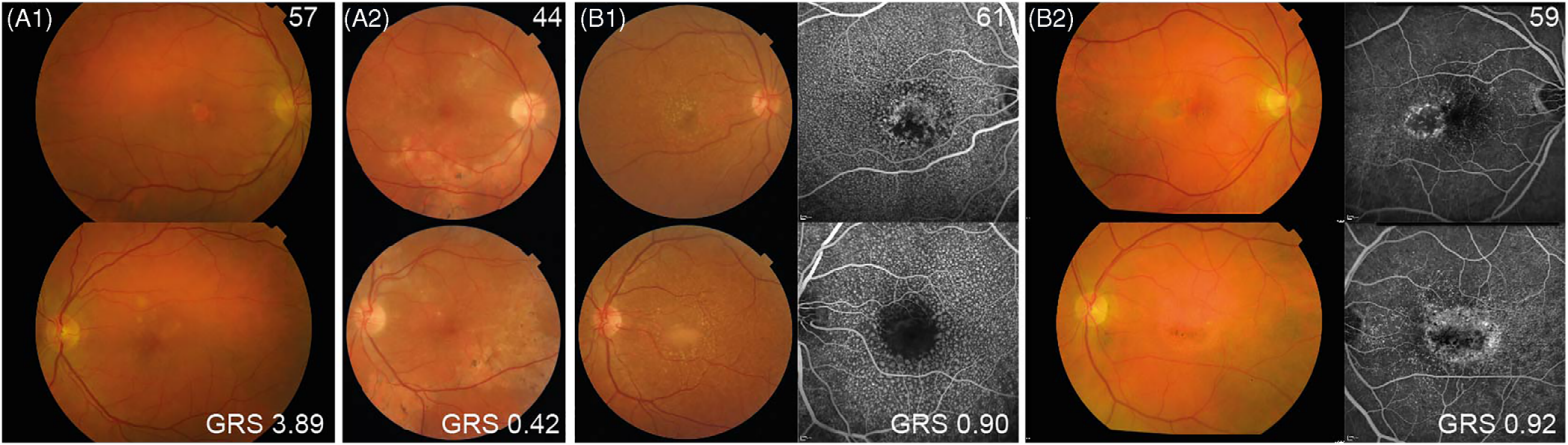
EODM Patients Carrying the Same Rare Variant in *LDLR* and *ACAT1*. (A) *LDLR* c.47 T > C (p.Leu16Pro) carriers. (A1) Patient with a central GA lesion (right eye) and intermediate/large drusen (left eye). (A2) Patient with small drusen, RPE alterations and atrophic areas (both eyes). (B) *ACAT1* c.1217A > G (p.Glu406Gly) carriers. (B1,B2) Numerous cuticular drusen spread across the posterior pole (both eyes) on colour fundus photographs and fluorescein angiography. Upper right corner = age; Bottom right corner = GRS; GRS, genetic risk score [Colour figure can be viewed at wileyonlinelibrary.com]

Besides the genes in before‐mentioned three candidate gene lists, we also analysed 633 genes located in 34‐AMD associated loci. In total, 93 rare variants in 76 candidate genes were identified in at least two EODM patients. Noteworthy, five genes were present in multiple candidate gene lists (*ELANE, LDLR, APOB, HTRA1, CFHR5*). Of these 93 overlapping rare variants, the majority (84/93) was identified in genes in the AMD‐associated loci, whereas the remaining nine overlapping variants were identified in complement‐related genes (*C1S, CSMD2, CFHR5, CR1, ELANE*), lipid‐related genes (*LDLR, APOB, ACAT1*) or in genes evaluated in mouse models that developed drusen or other AMD characteristics (*LDLR, APOB, HTRA1*). Based on variant type, in silico prediction tools and pathogenicity category according to ClinVar, we identified 20 rare variants that are potentially damaging (Table S4 section D, bold). Two of these variants, both identified in lipid‐related genes, stood out. The c.47 T > C (p.Leu16Pro) variant in *LDLR* is predicted to be pathogenic based on in silico prediction tools, and was reported in patients with familial hypercholesterolemia.^26^ The other rare variant (c.1217A > G [p.Glu406Gly]) in *ACAT1* was predicted to be pathogenic based on all three in silico prediction tools, however, in ClinVar it was reported as a variant of uncertain significance. No phenotypic overlap was observed between EODM patients carrying the *LDLR* c.47 T > C (p.Leu16Pro) variant (Figure 3A). Notably, patient A1 also suffers from C3 glomerulopathy, and shows signs of both EODM and central serous chorioretinopathy. The two EODM patients carrying the *ACAT1* c.1217A > G (p.Glu406Gly) variant presented with cuticular drusen, and in the left eye of patient B1 a pseudovitelliform lesion was observed (Figure 3B).

### 3.5 Evaluation of 18 IRD genes

Overlap in phenotypic characteristics between IRD and AMD has previously been reported.^21^ To investigate whether EODM may be a phenocopy from Mendelian macular dystrophies, we screened 18 IRD genes to rule out potential misdiagnoses. In total, 44 rare nonsense, frameshift, protein‐altering or splice‐site variants were identified in nine different IRD genes (Table S5). None of the EODM patients carried biallelic variants in the six genes described to cause AR IRDs, and none of the copy number variants or structural variants were identified in the EODM cohort. We evaluated the available imaging in detail for 11 variants identified in the *ABCA4, ABCC6, FSCN2, PRDM13, TIMP3* and *IMPG1* genes, as they were predicted to be pathogenic based on all three in silico prediction tools, or they were reported to be (likely) pathogenic in ClinVar or in literature (Table S6). Retinal images of the patients carrying potential pathogenic variants showed drusen in combination with other fundus features, such as RPE alterations, GA and CNV, which are characteristic for AMD, and not for IRD (Figure S1). All rare variants identified in candidate genes in EODM patients are listed in Table S7. Potential deleterious rare variants in non‐candidate genes are depicted in Table S8.

## 4 DISCUSSION

The present study comprehensively analysed the role of common and rare variants in EODM. We showed that the 52 AMD‐associated variants, and in particular the complement‐related variants, contributed to EODM development. Furthermore, several rare, potential deleterious variants in genes of the complement and lipid metabolism pathways, and genes evaluated in mouse models that developed drusen or other AMD characteristics, were identified that may play a role in the pathogenesis of EODM. In addition, some EODM patients carried rare potential deleterious variants in genes previously associated with IRDs mimicking AMD. However, evaluation of the patients' phenotype based on retinal imaging did not show an IRD phenotype.

To date, the aetiology of EODM is not fully understood. In fact, it is even not completely clear whether EODM is an early manifestation of AMD, or it is a separate disease entity caused by highly penetrant variants that act in an autosomal dominant manner. Its aetiology could also be a combination of both, where common AMD‐associated variants in combination with highly penetrant rare variants result in the development of AMD characteristics at an early age. Based on findings of our current study we propose the latter, with contributions of common and rare variants in the aetiology of EODM.

Regarding the contribution of common variants to EODM, we observed that especially complement‐related variants are involved in EODM, and complement‐related variants seem to contribute most in advanced disease stages, although this latter warrants further research in larger EODM cohorts. Our findings are in line with previous literature, in which a higher complement GRS was observed in advanced AMD stages,^27^ and higher systemic complement activation levels in advanced AMD stages were reported.^28^ Lipid‐related variants did not contribute to EODM in this current study, although we did observe a slightly higher mean lipid GRS in EODM and a trend of decreasing lipid GRS with increasing EODM disease stage. In a recent study by Thee et al. (in preparation), the authors observed that several systemic lipid and lipoprotein measurements were associated with the early/intermediate AMD stages.

With respect to rare variants, we did not identify specific rare variants that were present in a large proportion of EODM patients or in one specific gene. Most of the potential deleterious rare variants were found in a single or in two EODM patients. Several potential deleterious rare variants in specific genes identified in this current study are of interest. One of these variants includes *LDLR* c.47 T > C (p.Leu16Pro), which was identified in two EODM patients. The low‐density lipoprotein receptor (LDLR) is expressed in the RPE, and is localised in the basement membrane of the RPE. It is involved in the uptake of LDL particles by RPE cells from the circulation, and thereby belongs to the lipid pathway.^16^, ^18^ In a previous study, it was shown that lipid particles accumulated in Bruch's membrane in *Ldlr* deficient mice, emphasising the potential role of *LDLR* in AMD.^29^ Furthermore, variants in *LDLR*, including the c.47 T > C (p.Leu16Pro) variant, are associated with familial hypercholesterolemia.^26^ To the best of our knowledge, there is no direct link between AMD/EODM and hypercholesterolemia. Taken together, variants in *LDLR* might play a role in EODM. Further research is needed to elucidate the role of rare *LDLR* variants, e.g., evaluating lipid deposition in iPSc‐based AMD/EODM model systems, or measuring cholesterol levels in blood of patients with rare variants in *LDLR*. Although two EODM patients carried the same rare variant in *LDLR*, their phenotypes did not show similarities. Of interest, one of those patients suffered from C3 glomerulopathy. It is described before that patients with C3 glomerulopathy can develop retinal drusen, and part of the patients with C3 glomerulopathy carry risk alleles in complement‐related genes.^30^, ^31^, ^32^ This particular patient did not carry (likely) pathogenic rare variants in complement genes, but the complement GRS was relative high.

We also identified potential deleterious variants in two genes evaluated in mouse models that developed AMD characteristics, including *CD36* and *CX3CR1*. CD36 is expressed in the RPE and involved in the uptake of lipoproteins. One EODM patient carried a rare nonsense variant (c.1144C > T [p.Gln382*]) and one a rare missense variant (c.31G > A [p.Ala11Thr]) *CD36*. Although this latter variant was not predicted to be deleterious, the first one results in a premature stop codon and is predicted to be damaging (CADD 47.0, Grantham 1000). Moreover, a previous study showed age‐related photoreceptor degeneration and choroidal involution in *Cd36* deficient mice, pointing towards a potential important role of *CD36* in the pathogenesis of EODM.^33^ Two other rare variants were identified in *CX3CR1* in two EODM patients. *Cx3CR1 c*.74A > G (p.Asp25Gly) is predicted to be pathogenic based on all three in silico prediction tools. The functional effect of the other *CX3CR1* variant (c.169A > G [p.Thr57Ala]) is unclear. The CX3C chemokine receptor 1 (CX3CR1) is expressed in retinal microglial cells. In a previous study, lipid‐bloated microglial cells accumulated in the subretinal space of *Cx3cr1* knockout mice. Furthermore, microglial cell accumulation in these mice was associated with an exacerbation of laser‐induced CNV. In addition, other AMD characteristics, including complement deposition and photoreceptor atrophy, were observed in *Cx3cr1* knockout mice, emphasising the potential role of this gene in EODM.^20^, ^34^ The patient carrying the (c.74A > G (p.Asp25Gly)) variant presented with drusen in both eyes and a CNV in the right eye. The identified variant might contribute to the EODM phenotype in this patient.

Furthermore, multiple nonsense, stop‐loss and frameshift variants were identified in EODM patients in genes of the alternative complement pathway, a pathway that has firmly been confirmed to play an important role in AMD pathogenesis. Based on variant type, these variants are considered to be pathogenic, however, one needs to take into account the effect of the variants on the specific proteins. For example, the factor H related (FHR) proteins compete with factor H (FH) in binding to C3b, thereby counteracting complement inhibition by FH. Less inhibition by FH results in increased complement activation, which in turn is a risk factor for AMD. Therefore, nonsense variants in *CFHR* genes are considered to be protective for AMD, rather than risk increasing. This is in line with a recent study, reporting that low‐frequency variants resulting in low or absent levels of FHR‐2 and FHR‐5 are protective for AMD.^35^ The nonsense variant identified in the EODM patient in this study (c.595G > T [p.Glu199*]), was reported to lead to complete absence of FHR‐2.^36^ Another rare variant located at the C‐terminus in the last exon of *C3* (c.4992A > G (p.Ter1664TrpextTer24)), was identified in one EODM patient, and was not reported in AMD before. This variant is predicted to add 24 erroneous amino acids to the C‐terminus of C3, and could potentially interfere with correct folding of C3, and likely results in degradation within the cell. However, functional data for this variant is lacking. Noteworthy, this particular patient has a high GRS, and carries multiple risk alleles in complement‐related genes, which may contribute to the patients' phenotype. Furthermore, two nonsense variants and one frameshift variant in *C8A*, *C8B* and *C9* were identified. These three genes encode components of the membrane attack complex, which plays an important role in targeting pathogens. In general, mutations (especially nonsense mutations) in the C5–C9 genes are associated with different types of C5–C9 deficiencies (e.g. C9 deficiency in the Japanese population).^37^, ^38^ Higher complement activation levels are reported in AMD patients compared to control individuals.^28^ Therefore, we expect that nonsense mutations in *C5*–*C9* are protective for AMD, and presumably also for EOMD, considering the same underlying disease mechanisms. Nevertheless, it is important to note that EODM is likely due to a combination of risk increasing and risk decreasing genetic variants. Last, in one patient we identified a splice‐site variant in *CFH* (c.350 + 6 T > G). In a previous study it was suggested that monogenic inheritance of *CFH* variants could result in drusen at a young age.^7^ In the patient in which we identified the c.350 + 6 T > G variant, we did not find any other rare variants in *CFH*. However, the GRS in this patient was high (2.03). The high genetic load together with the rare variant in *CFH* fits with the EODM phenotype is this patient.

Awareness for overlap in phenotypic features between AMD/EODM and IRDs is warranted, especially in patients presenting with drusen or other AMD characteristics at an early age. In this study we identified one patient with a *TIMP3* c.70 T > G (p.Cys24Gly) mutation. Mutations in *TIMP3* are linked to SFD, an IRD characterised by CNV with or without the presence of drusen. It is reported that most of the disease‐causing mutations in *TIMP3* involve cysteine residues.^39^ The patient carrying a rare variant in *TIMP3* in this current study, presented with large drusen and small (nascent) GA lesions, which was not suspect for SFD. The GRS was high (2.53), suggesting that the disease may be primarily driven by AMD‐associated variants. However, the *TIMP3* variant might additionally contribute to the patients' phenotype. Phenotypic characteristics of the other EODM patients in whom we identified a rare variant associated with an IRD did not point towards an IRD. We cannot confirm nor rule out that the identified variants contribute to the patients' phenotype. For future studies it can be helpful to include family members, since this could provide information about segregation of specific genetic variants.

To our knowledge, this is the first study using WGS in a large cohort of EODM patients, and allowed for the evaluation of both common and rare variants, and revealed insights into biological disease pathways that may contribute to EODM. This study also has its limitations. The limited sample size and the design of the study did not allow to detect significant associations of rare variants with EODM. However, we identified several rare variants in EODM patients that are potentially involved in EODM. These findings should be replicated in a larger cohort of EODM patients, to elucidate their contribution to EODM. Furthermore, many variants were not reported previously, were of uncertain clinical significance, or were predicted to be (likely) pathogenic based on in silico prediction tools. Testing the effect of these variants in AMD/EODM model systems (e.g. mouse or iPSc‐based models), or by measuring protein or metabolite levels in patients with specific rare variants, will improve the interpretation of these variants and may result in new insights in the EODM pathogenesis. In the current study family members of EODM patients were unfortunately not available, therefore, we recommend ascertaining family members for segregation analysis in a follow‐up study. We consider EODM as an early manifestation of AMD, which is a multifactorial disease. In some EODM cases a high GRS based on the AMD‐associated variants may already explain the disease. In other cases where the GRS is low and where we did not find potential deleterious rare variants in the studied candidate genes, the cause may lie in other genomic regions or in genes that were not evaluated in this study. In addition, we focused solely on genetic factors, and did not analyse non‐genetic risk factors such as age, smoking and diet, as we hypothesised that EODM is more genetically driven compared to AMD due to the early age of onset.

## 5 CONCLUSION

In this current study we showed that common AMD‐associated variants contribute to EODM development, and we identified rare, potential deleterious variants that might play a role in EODM pathogenesis. Together with the identification of rare *CFH* variants in a large proportion of EODM patients,^11^ we consider EODM as an early manifestation of AMD, in which both common and rare variants contribute to its pathogenesis.

## AUTHOR CONTRIBUTIONS

Anita de Breuk, Carel B. Hoyng, and Anneke I. den Hollander: Concept and design; All authors: acquisition, analysis, or data interpretation; Anita de Breuk and Anneke I. den Hollander: Manuscript drafting; All authors: critical revision of the manuscript for important intellectual content; Anita de Breuk and Anneke I. den Hollander: statistical analysis; Caroline C. W. Klaver, Carel B. Hoyng, and Anneke I. den Hollander, and Anita de Breuk: Obtained funding; Yara T. E. Lechanteur, Caroline C. W. Klaver, Carel B. Hoyng, and Anneke I. den Hollander: Supervision.

## FUNDING INFORMATION

This research was supported by the Dutch Research Council (016.Vici.170.024 to Anneke I. den Hollander) and by the Bayer Ophthalmology Research Award (BORA). The funding organizations had no role in the design or conduct of the study and were not involved in interpretation of the data or writing of the manuscript.

## CONFLICT OF INTEREST

Anita de Breuk, Yara T. E. Lechanteur, Galuh Astuti, Jordi Corominas Galbany, Caroline C. W. Klaver, and Carel B. Hoyng declare no conflict of interest. Anneke I. den Hollander is a consultant for Gemini Therapeutics, Gyroscope Therapeutics, Ionis Pharmaceuticals and Roche.

## ETHICS STATEMENT

This research was approved by ethical committees at the Radboud university medical centre, Nijmegen, The Netherlands, the University Hospital of Cologne, Cologne, Germany, and the Institut de la Màcula, Barcelona, Spain, and adhered to the tenets of the Declaration of Helsinki, and all study participants provided written informed consent. Approval code 2007‐158. Approval date whole genome sequencing, August 21st, 2018.

## Supporting information


**Figure S1** Colour Fundus Photographs of EODM Patients Carrying Rare Variants in IRD Genes

**Table S1** Overview of 52 AMD‐associated variants identified in a large GWAS study (adapted from Fritsche et al., 2016)

**Table S2** Candidate Gene Lists

**Table S3** Genetic risk scores per patient

**Table S4** Candidate genes and variants

**Table S5** Rare variants in inherited retinal dystrophy genes

**Table S6** Rare Variants in IRD genes predicted to be damaging by three prediction algorithms or reported as (likely) pathogenic by ClinVar

**Table S7** All rare variants in candidate genes in the 49 EODM patients

**Table S8** Nonsense, frameshift, splice‐site and missense variants with high prediction score

## Data Availability

The data that supports the findings of this study are available in the supplementary material of this article.
