# Supplementary material for: Common and rare variants in patients with early onset drusen maculopathy
Source: Clin Genet. 2022 Sep 13;102(5):414–23. doi: 10.1111/cge.14212 (PMC9825904; doi:10.1111/cge.14212)
Supplement: Supplementary file 1 — Figure S1 Colour Fundus Photographs of EODM Patients Carrying Rare Variants in IRD Genes [file CGE-102-414-s005.docx]

**Supporting Information Figure S1.** Colour Fundus Photographs of EODM Patients Carrying Rare Variants in IRD Genes


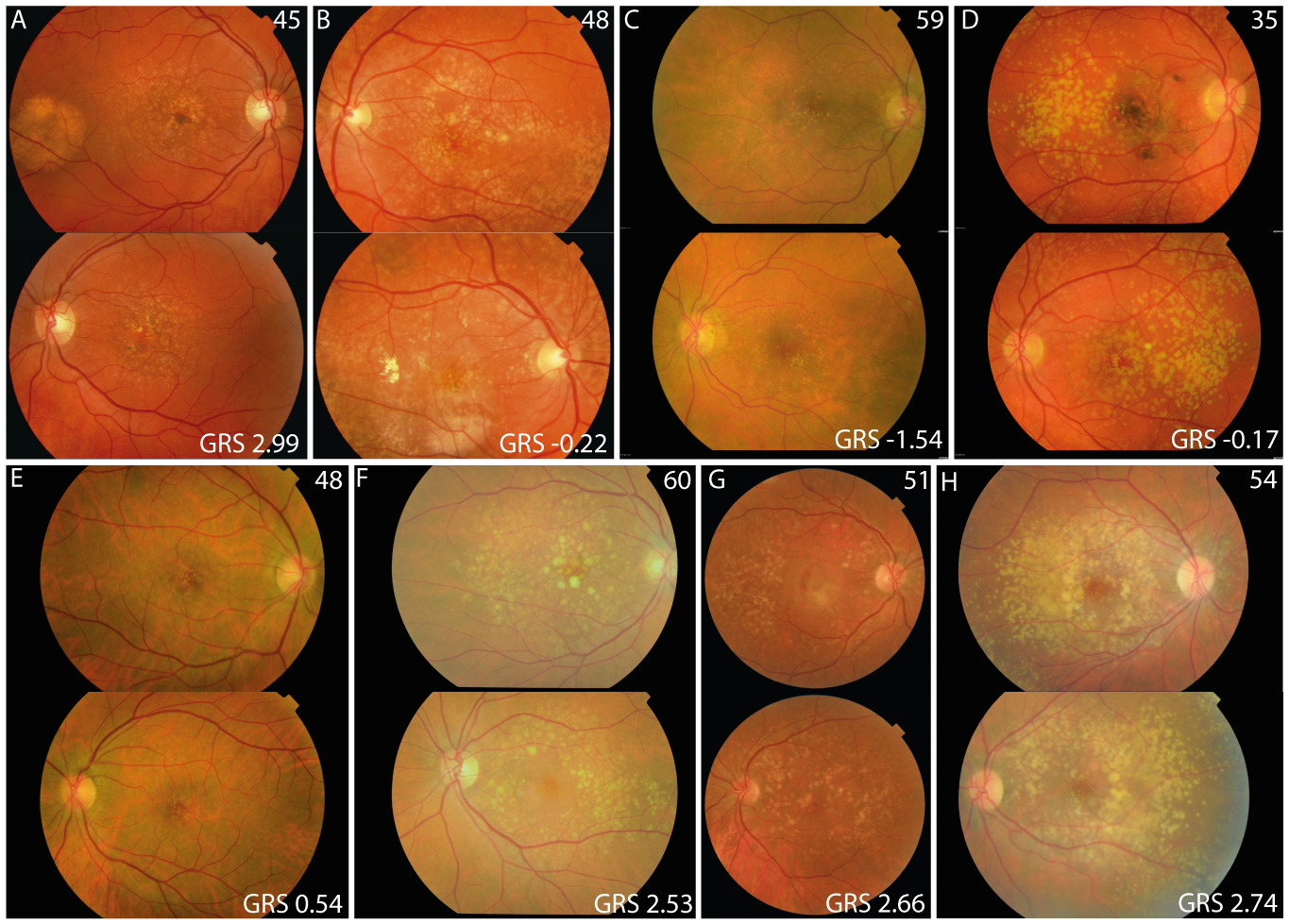


Colour fundus photographs of patients with EODM carrying potential deleterious rare protein-altering or splice-site variants in inherited retinal dystrophy genes that can mimic AMD. Identified variants and phenotypic descriptions of the patients are depicted in Supporting Information Table S6. Number in the right upper corner of each panel represent the age of the patients. Genetic risk scores are indicated in the right lower corner of each panel. GRS = genetic risk score.
