# Supplementary material for: Common and rare variants in patients with early onset drusen maculopathy
Source: Clin Genet. 2022 Sep 13;102(5):414–23. doi: 10.1111/cge.14212 (PMC9825904; doi:10.1111/cge.14212)
Supplement: Supplementary file 2 — Table S1 Overview of 52 AMD‐associated variants identified in a large GWAS study (adapted from Fritsche et al., 2016) [file CGE-102-414-s003.docx]

**Supporting Information Table S1.** Overview of 52 AMD-associated variants identified in a large GWAS study (adapted from Fritsche et al., 2016)

| **Chr** | **Position** | **Gene** | **Rs number** | **OR** | **Effect size** |
| --- | --- | --- | --- | --- | --- |
| 1 | 196,704,632 | *CFH* | 10922109 ^†^ | 0.51 | -0.67 |
| 1 | 196,657,064 | *CFH* | 570618 ^†^ | 1.74 | 0.55 |
| 1 | 196,716,375 | *CFH* | 121913059 ^†^ | 47.63 | 3.86 |
| 1 | 196,613,173 | *CFH* | 148553336 ^†^ | 0.31 | -1.17 |
| 1 | 196,380,158 | *CFH* | 187328863 ^†^ | 1.47 | 0.39 |
| 1 | 196,815,450 | *CFH (CFHR3/CFHR1)* | 61818925 ^†^ | 1.18 | 0.17 |
| 1 | 196,706,642 | *CFH* | 35292876 ^†^ | 1.54 | 0.43 |
| 1 | 196,958,651 | *CFH* | 191281603 ^†^ | 0.41 | -0.89 |
| 2 | 228,086,920 | *COL4A3* | 11884770 | 0.92 | -0.08 |
| 3 | 64,715,155 | *ADAMTS9-AS2* | 32247658 | 1.14 | 0.13 |
| 3 | 99,180,668 | *COL8A1* | 140647181 | 1.85 | 0.62 |
| 3 | 99,419,853 | *COL8A1* | 55975637 | 1.16 | 0.15 |
| 4 | 110,659,067 | *CFI* | 10033900 ^†^ | 1.15 | 0.14 |
| 4 | 110,685,820 | *CFI* | 141853578 ^†^ | 5.12 | 1.63 |
| 5 | 39,327,888 | *C9* | 62358361 ^†^ | 1.67 | 0.51 |
| 5 | 35,494,448 | *PRLR/SPEF2* | 114092250 | 0.71 | -0.34 |
| 6 | 31,930,462 | *C2/CFB/SKIV2L* | 116503776 ^†^ | 0.51 | -0.67 |
| 6 | 31,946,792 | *C2/CFB/SKIV2L* | 144629244 ^†^ | 2.79 | 1.03 |
| 6 | 32,155,581 | *C2/CFB/SKIV2L (PBX2)* | 114254831 ^†^ | 1.13 | 0.12 |
| 6 | 31,947,027 | *C2/CFB/SKIV2L* | 181705462 ^†^ | 1.56 | 0.45 |
| 6 | 43,826,627 | *VEGFA* | 943080 | 0.87 | -0.14 |
| 7 | 104,756,326 | *KMT2E/SRPK2* | 1142 | 1.14 | 0.13 |
| 7 | 99,991,548 | *PILRB/PILRA* | 7803454 | 1.15 | 0.14 |
| 8 | 23,082,971 | *TNFRSF10A* | 79037040 | 0.89 | -0.12 |
| 9 | 76,617,720 | *MIR6130/RORB* | 10781182 | 1.12 | 0.11 |
| 9 | 73,438,605 | *TRPM3* | 71507014 | 1.11 | 0.10 |
| 9 | 101,923,372 | *TGFBR1* | 1626340 | 0.88 | -0.13 |
| 9 | 107,661,742 | *ABCA1* | 2740488 ^‡^ | 0.89 | -0.12 |
| 10 | 24,999,593 | *ARHGAP21* | 12357257 | 1.12 | 0.11 |
| 10 | 124,215,565 | *ARMS2/HTRA1* | 3750846 | 2.93 | 1.08 |
| 12 | 56,115,778 | *RDH5/CD63* | 3138141 | 1.18 | 0.17 |
| 12 | 112,132,610 | *ACAD10* | 61941274 | 1.60 | 0.47 |
| 13 | 31,821,240 | *B3GALTL* | 9564692 | 0.90 | -0.11 |
| 14 | 68,769,199 | *RAD51B* | 61985136 | 0.88 | -0.13 |
| 14 | 68,986,999 | *RAD51B* | 2842339 | 1.18 | 0.17 |
| 15 | 58,680,954 | *LIPC* | 2043085 ^‡^ | 1.15 | 0.14 |
| 15 | 58,723,939 | *LIPC* | 2070895 ^‡^ | 0.86 | -0.15 |
| 16 | 56,997,349 | *CETP* | 5817082 ^‡^ | 0.87 | -0.14 |
| 16 | 56,994,528 | *CETP* | 17231506 ^‡^ | 1.11 | 0.10 |
| 16 | 75,234,872 | *CTRB2/CTRB1* | 72802342 | 0.79 | -0.24 |
| 17 | 26,649,724 | *TMEM97/VTN* | 11080055 ^†^ | 0.92 | -0.08 |
| 17 | 79,526,821 | *NPLOC4/TSPAN10* | 6565597 | 1.12 | 0.11 |
| 19 | 6,718,387 | *C3* | 2230199 ^†^ | 1.47 | 0.39 |
| 19 | 6,718,146 | *C3* | 147859257 ^†^ | 3.22 | 1.17 |
| 19 | 5,835,677 | *C3 (NRTN/FUT6)* | 12019136 ^†^ | 0.74 | -0.30 |
| 19 | 1,031,438 | *CNN2* | 67538026 | 0.90 | -0.11 |
| 19 | 45,441,941 | *APOE* | 429358 ^‡^ | 0.67 | -0.40 |
| 19 | 45,748,362 | *APOE (EXOC3L2/MARK4)* | 73036519 ^‡^ | 0.91 | -0.09 |
| 20 | 44,614,991 | *MMP9* | 142450006 | 0.84 | -0.17 |
| 20 | 56,653,724 | *C20orf85* | 201459901 | 0.76 | -0.27 |
| 22 | 33,105,817 | *SYN3/TIMP3* | 5754227 | 0.79 | -0.24 |
| 22 | 38,476,276 | *SLC16A8* | 8135665 | 1.14 | 0.13 |

The 52 AMD-associated variants identified in a large genome-wide association study (adapted from Fritsche et al., 2016)^2^ used for calculation of the genetic risk score. † Variants included in the complement GRS. ‡ Variants included in the lipid GRS. Chr = chromosome; OR = odds ratio
