# Supplementary material for: Common and rare variants in patients with early onset drusen maculopathy
Source: Clin Genet. 2022 Sep 13;102(5):414–23. doi: 10.1111/cge.14212 (PMC9825904; doi:10.1111/cge.14212)
Supplement: Supplementary file 7 — Table S6 Rare Variants in IRD genes predicted to be damaging by three prediction algorithms or reported as (likely) pathogenic by ClinVar [file CGE-102-414-s004.docx]

**Supporting Information Table S6.** Rare Variants in IRD genes predicted to be damaging by three prediction algorithms or reported as (likely) pathogenic by ClinVar

| **Variant** | **Disease association** | **PhyloP** | **CADD** | **Grantham** | **Pathogenicity** (ClinVar) | **EODM patients** (n, MAF %) | **gnomAD** v2.1.1. (MAF %) | **Evaluation phenotypic characteristics on retinal imaging** |
| --- | --- | --- | --- | --- | --- | --- | --- | --- |
| **Genes associated with AR macular dystrophies** | | | | | | | | |
| *ABCA4* c.6089G>A (p.Arg2030Gln) | Stargardt disease | 6.0 | 31.0 | 43 | Pathogenic/Likely pathogenic | 1 (1.02%) | 0.04 | 35-year-old female with large soft, confluent, indistinct drusen extending to the temporal and peripheral retina in both eyes. Retinal pigment epithelium alterations are present in the center of the macula (*Supplementary Figure 1, panel D*). |
| *ABCA4* c.3113C>T (p.Ala1038Val) |  | 7.9 | 22.4 | 64 | Pathogenic/Likely pathogenic | 1 (1.02%) | 0.18 | 59-year-old female with intermediate sized drusen and retinal pigment epithelium alterations in the posterior pole of both eyes (*Supplementary Figure 1, panel C*). |
| *ABCA4* c.2267C>T (p.Ser756Phe) ^†^ |  | 6.2 | 23.8 | 155 | VUS | 1 (1.02%) | 0.006 | 51-year-old female with large soft indistinct drusen in both eyes. The right eye developed a CNV (*Supplementary Figure 1, panel G*). ^†^ |
| *ABCA4* c.4253+43G>A |  | - | - | - | VUS * | 1 (1.02%) | 0.47 | 54-year-old female with large confluent soft drusen and several small areas of atrophy in both eyes (*Supplementary Figure 1, panel H*). |
| *ABCC6* c.3979G>A (p.Gly1327Arg) ^‡^ | Pseudoxanthoma elasticum | 4.0 | 25.2 | 125 | NA | 1 (1.02%) | 0.01 | 60-year-old female with large drusen and several small (nascent) GA lesions (*Supplementary Figure 1, panel F*). ^‡^ |
| *ABCC6* c.3389C>T (p.Thr1130Met) ^§^ |  | 4.8 | 27.3 | 81 | Pathogenic/Likely pathogenic | 1 (1.02%) | 0.007 | 48-year-old male with multiple drusen located in the macula of both eyes (*Supplementary Figure 1, panel E*). ^§^ |
| **Genes associated with AD macular dystrophies** | | | | | | | | |
| *FSCN2* c.412C>T (p.His138Tyr) ^§^ | AD macular degeneration,  AD RP | 7.2 | 26.2 | 83 | Benign | 1 (1.02%) | 0.72 | § |
| *PRDM13* c.863A>C (p.Tyr288Ser) ^†^ | North-Carolina macular dystrophy | 4.5 | 27.1 | 144 | NA | 1 (1.02%) | NA | † |
| *PRDM13* c.1086_1088del (p.His363del) |  | 3.2 | 25.1 | 1000 | NA | 1 (1.02%) | NA | 48-year-old female with drusen in both eyes, and a CNV with hard exsudates in the right eye (*Supplementary Figure 1, panel B*). |
| *TIMP3* c.70T>G (p.Cys24Gly) ^‡^ | Sorsby fundus dystrophy | 5.1 | 29.5 | 159 | NA | 1 (1.02%) | NA | ‡ |
| **Genes associated with AR or AD macular dystrophies** | | | | | | | | |
| *IMPG1* c.2243G>T (p.Arg748Met) | AD benign concentric annular macular dystrophy, AD and AR vitelliform macular dystrophies | 4.9 | 29.0 | 91 | NA | 1 (1.02%) | NA | 45-year-old female with drusen and retinal pigment epithelium alterations in the macula of both eyes (*Supplementary Figure 1, panel A*). |

Potential damaging rare variants identified in EODM patients in genes previously associated with inherited autosomal recessive (AR) and/or autosomal dominant (AD) macular dystrophies that can mimic AMD/EODM characteristics. Potential deleteriousness based on *in silico* prediction tools was based on the following thresholds: PhyloP ≥ 2.7, CADD ≥ 15, Grantham score ≥ 80. †, ‡, § represent three EODM patients carrying two different variants. * Deep intronic variant, in ClinVar conflicting results regarding the pathogenicity. In Stargardt disease this variant was reported to result in skipping of exon 28 (PMID: 30643219); EODM = early onset drusen maculopathy; IRD = inherited retinal dystrophy; CADD = Combined Annotation Dependent Depletion; gnomAD = genome aggregation database; AD = autosomal dominant; AR = autosomal recessive; RP = retinitis pigmentosa; GA = geographic atrophy; CNV = choroidal neovascularization.
